# Serological Evidence of Soil‐Transmitted Helminth Infections as a Potential Risk for Severity in Leprosy Patients

**DOI:** 10.1111/tmi.70020

**Published:** 2025-08-16

**Authors:** Ana Laura Grossi de Oliveira, Augusto César Parreiras de Jesus, Ramayana Morais de Medeiros Brito, Jordânia Costa‐Pinto, Tatyane Martins Cirilo, José Bryan Rihs, Marcelo Eduardo Cardozo, Lilian Lacerda Bueno, Luisa Mourão Dias Magalhães, Ricardo Toshio Fujiwara

**Affiliations:** ^1^ Laboratory of Immunobiology and Control of Parasites, Department of Parasitology, Institute of Biological Sciences Federal University of Minas Gerais Belo Horizonte Minas Gerais Brazil; ^2^ Postgraduate Program in Health Sciences: Infectious Diseases and Tropical Medicine, Faculty of Medicine Federal University of Minas Gerais Belo Horizonte Minas Gerais Brazil; ^3^ Sensor Engineering Department, Faculty of Science and Engineering Maastricht University Maastricht Limburg the Netherlands; ^4^ Postgraduate Program in Parasitology, Biological Science Institute Federal University of Minas Gerais Belo Horizonte Minas Gerais Brazil; ^5^ Laboratory of Interactions in Immuno‐Parasitology, Department of Parasitology, Institute of Biological Sciences Federal University of Minas Gerais Belo Horizonte Minas Gerais Brazil

**Keywords:** co‐infections, household contacts, IgG seroprevalence, leprosy, leprosy reactions, soil‐transmitted helminths

## Abstract

Leprosy presents a broad clinical spectrum influenced by the host's immune response, and co‐infections may further modulate disease progression. This study evaluated clinically diagnosed leprosy patients (*n* = 251) from Sergipe and Minas Gerais, Brazil, along with healthy controls (*n* = 43), soil‐transmitted helminths‐positive controls (*n* = 15), and household contacts (*n* = 176). Enzyme‐linked immunosorbent assays were performed using predicted B‐cell epitopes from immunogenic proteins of *Ascaris* sp., *Trichuris trichiura*, *Strongyloides stercoralis*, *Ancylostoma duodenale* and *Necator americanus* . Among leprosy patients, 123 (49%) were IgG seropositive for at least one soil‐transmitted helminths antigen. Nine (7.3%) had optical density (OD) values exceeding 2‐fold the cut‐off, six (4.9%) surpassed 3‐fold and one exceeded 7‐fold. In patients with documented reactions (*n* = 34), seropositivity was observed in 6 with neuritis, 14 with type 1 reaction (T1R) and 14 with type 2 reaction (T2R), totaling 61.8%. Among household contacts, 96 (54.5%) were seropositive. Stratified analyses revealed significant differences in IgG levels between soil‐transmitted helminths‐seropositive and seronegative individuals within both paucibacillary and multibacillary groups, despite no overall association with operational classification. These findings suggest that helminth exposure may influence immune responses within leprosy subtypes and contribute to reactional episodes. The high seroprevalence observed in both patients and household contacts highlights shared environmental exposure and supports the inclusion of helminth monitoring in leprosy control strategies. Early detection and treatment of co‐infections may reduce immune imbalances and severe inflammatory outcomes. Further studies are needed to elucidate the immunological mechanisms underlying helminth–leprosy interactions and to strengthen integrated approaches in public health.

## 1 Introduction

Leprosy is a chronic infectious disease caused by *Mycobacterium leprae* and *Mycobacterium lepromatosis*, characterised by a wide clinical spectrum that reflects the host's innate, cellular and humoral immune responses [1, 2, 3]. Parasitic co‐infections, particularly with soil‐transmitted helminths (STHs), affect the most vulnerable populations in the world [4] and co‐occur in regions where leprosy is also endemic [5]. Despite being both preventable and treatable, STHs and leprosy cause an estimated 50 million disability‐adjusted life years (DALYs) and stigmatisation worldwide, with negative social and economic consequences for those infected [6, 7, 8]. Notably, genetic susceptibility and repeated or prolonged exposure to *M. leprae* have been strongly associated with disease development, which may help explain the ongoing transmission of leprosy in high‐risk populations despite the availability of effective treatment [9, 10, 11].

Polyparasitism modulates immune dynamics and can alter how these diseases are managed [12, 13]. STH infections can skew the immune response towards a Th2 profile, potentially compromising the host's ability to build an effective defence against *M. leprae* [14]. In addition, helminth co‐infection can suppress the immune response [15], alter the host microbiota after treatment [16] and compromise immune surveillance, which affects leprosy prognosis [17]. Importantly, immunosuppressive therapies used to treat leprosy reactions, particularly corticosteroids, can lead to fatal hyperinfection in patients harbouring undiagnosed *Strongyloides stercoralis* [18].

Leprosy combined with other parasitic infections can significantly alter the humoral response, such as IgG production [15, 19, 20, 21], either through immune suppression [13] or immune signalling disruption [14]. Understanding how polyparasitism affects the immune system and the progression of infectious diseases such as leprosy is critical to improving management [22]. As neglected tropical diseases (NTDs), their control will contribute to the achievement of the 3.3 target of the United Nations Sustainable Development Goals [23]; therefore, effective control strategies should address both leprosy and parasitic co‐infections because targeting only one of these factors may result in incomplete or suboptimal outcomes [24].

Despite the well‐recognised impact of co‐infections on immune modulation, the specific interplay between STH infections, leprosy clinical manifestations and reactional episodes remains poorly understood. In this context, the present study investigated the seroprevalence of IgG antibodies against STH‐predicted peptides in individuals with leprosy to better understand how these co‐endemic infections may influence immune dynamics. Identifying co‐infection or co‐exposure to helminths and *M. leprae* is essential to prevent potential immune responses that could negatively affect leprosy prognostic and clinical management, particularly in regions where both diseases are endemic.

## 2 Materials and Methods

### 2.1 Ethical Considerations

The present study was approved by the Ethics Committees of the Federal University of Minas Gerais (#14887414.0.0000.5149) and the Federal University of Sergipe (#0152.0.107.000‐07). Leprosy patients and household contacts voluntarily signed an informed consent form in agreement.

### 2.2 Characteristics of Leprosy Patients, STH‐Positive Group and Healthy Subjects

Sera used in this study are from the serum repository of the Laboratory of Immunobiology and Control of Parasites from the Federal University of Minas Gerais and were previously described [19]. This repository contains serum samples from well‐characterised leprosy patients and control groups. Previously, a total of 251 leprosy patients under treatment were recruited at the Regional Reference Center for Infectious Diseases (FHEMIG), the René Rachou Research Center, FIOCRUZ Institute in Minas Gerais State and the Dermatology Clinic of the Federal University Hospital in Sergipe State. Leprosy patients were classified according to WHO criteria based on skin lesions as paucibacillary (PB) or multibacillary (MB) to ensure the inclusion and consistent analysis of the sample set. Inflammatory leprosy reactions were categorised as neuritis, type 1 reaction (T1R) and type 2 reaction (T2R). Additionally, leprosy household contacts (*n* = 176) from endemic areas were included as a comparative group for helminthiasis exposure.

STH‐positive samples from the MG endemic area were tested following WHO recommendations for the presence of helminth ova or larvae, using a Kato‐Katz smear to determine infection intensity [4]. Participants were classified as helminth‐positive (STH+) (*n* = 15) or helminth‐negative (STH−) (*n* = 43) based on parasitological and serological screening for helminthiasis.

### 2.3 Selection of B‐Cell Antigenic Epitopes Using Bioinformatics Tools

Proteomic data were obtained from the National Center for Biotechnology and the WormBase ParaSite electronic database (version WBPS10), which includes STH species such as *Ascaris* sp., hookworms (*Ancylostoma duodenale* and *Necator americanus* ), *Strongyloides stercoralis* and *Trichuris trichiura*. B‐cell epitopes were predicted from the proteins of each genome using Bepipred (for linear epitope prediction) and IUpred (for structural disorder prediction). The highest‐scoring epitopes for each helminth were selected, with identical sequences removed. Only epitopes conserved across all helminths were retained. Subsequently, genus‐level analyses were performed, and epitopes were selected to achieve 80% coverage and 100% identity, as assessed by BlastP. At each stage, duplicate epitopes or those of insufficient length were discarded, retaining only peptides of up to 15 amino acids. The predicted B‐cell peptides were then synthesised using the spot synthesis method on a cellulose membrane and tested for specific reactivity with sera from individuals infected with helminths and those who were uninfected. Peptide selection was based on recognition intensity, as previously described for diagnostic purposes [25]. Based on these criteria, 10 STH peptides were selected for soluble synthesis. The selected peptides were validated and patented at the Brazilian Institute of Industrial Property (INPI) under the patent number BR10 2023 027792 6 [26]. To minimise the possibility of cross‐reactivity, the selected peptide sequences were compared against known *Mycobacterium leprae* and *Mycobacterium lepromatosis* proteins using sequence alignment tools.

### 2.4 Immunoreactivity and Detection of Antigen‐Specific IgG

IgG serum reactivities were assessed using an in‐house enzyme‐linked immunosorbent assay (ELISA). Microtiter plates (96‐well; Corning, Merck, USA) were coated with 2 μg of a pool of 10 STH peptides diluted in carbonate buffer (pH 9.6) and incubated overnight at 4°C. Plates were washed five times with 0.05% PBS‐Tween 20 and blocked with 200 μL of PBS containing 5% BSA for 2 h at 37°C. After removing the blocking solution, 100 μL of serum (1:100 in PBS with 2.5% BSA), including controls or samples from leprosy patients, was added and incubated for 1 h at 37°C. Plates were washed again, followed by the addition of 100 μL of HRP‐conjugated anti‐human IgG (1:10,000 in PBS with 2.5% BSA; Sigma‐Aldrich, USA), and incubated for 1 h at 37°C. After a final wash, 50 μL of TMB substrate was added and incubated for 15 min at 37°C. The reaction was stopped with 25 μL of 0.2 M H_2_SO_4_. Optical density (OD) was measured at 450 nm using a VersaMax microplate reader (Molecular Devices, USA) and analysed with SoftMax Pro software v5.3. Cut‐off values were defined as the mean OD of negative control sera plus 3 standard deviations (SD). OD values ≥ 0.6092 nm were considered positive.

### 2.5 Statistical Analysis

GraphPad Prism (v.9.0) software (GraphPad Software, San Diego, CA, USA) was used for graph generation and statistical analysis. Data normality was assessed using the Shapiro–Wilk test. Parametric data were analysed using one‐way ANOVA, followed by Tukey's post hoc test, while non‐parametric data were analysed using the Kruskal‐Wallis test with Dunn's multiple comparisons post hoc test. To assess associations between categorical variables (e.g., helminth seropositivity and operational classification of leprosy), Fisher's exact test was applied. A *p* value < 0.05 was considered statistically significant for all tests.

## 3 Results

### 3.1 Main Characteristics

This study included 251 leprosy patients from the Brazilian states of Minas Gerais (MG; *n* = 154) and Sergipe (SE; *n* = 97), with a mean age of 45.4 years (range: 6–88 years). According to the WHO classification, 128 (83.1%) patients from MG and 51 (52.6%) from SE were classified as MB, while the remaining 72 (28.7%) were classified as PB. These baseline characteristics were similar to those reported in our previously published data [19].

Among these patients, information on inflammatory leprosy reactions (LR) was available for 55 (21.9%) individuals. Of these, 9 (16.4%) had neuritis, 27 (49.1%) had T1R and 19 (34.5%) had T2R. OD values equal to or higher than the cutoff (0.6092 nm) were observed in 34 (61.8%) patients in the LR group (Table 1).

**TABLE 1:** Main characteristics of the participants included in this study.

Leprosy (*n* = 251)	*n*	%	WHO leprosy classification	
			PB	MB
Minas Gerais state	154	61.3	26	128
Sergipe state	97	38.7	46	51
Total	251	100	72	179
Mean age (range)	45.4 (6–88 years)			

Inflammatory leprosy reaction type (*n* = 55)			STH IgG seropositivity OD readings (≥ 0.6092 nm)	
Neuritis	9	16.4	6	10.9
T1R	27	49.1	14	25
T2R	19	34.5	14	25
Total	55	100	34	100

Control groups consisted of 43 individuals from non‐endemic areas and 15 STH‐positive serum samples from adults in endemic areas (ages 27–36 years). Additionally, 176 household contacts of leprosy patients were included as a negative control group from the endemic area (MB contacts = 100; PB contacts = 76).

### 3.2 IgG Seropositivity to STH in Leprosy Patients and Control Groups

IgG seropositivity against STH antigens was assessed in a cohort of 251 leprosy patients and 234 individuals without leprosy (LP− = 58 and HHC = 176). The OD values obtained from each group were analysed and compared based on a calculated cut‐off value of 0.6092 to determine STH seropositivity. In the leprosy group, the seropositive individuals (*n* = 123) for STH (LP+/STH+) had OD values ranging from 0.6103 to 4.2584, which were significantly higher than those of STH seronegative (*n* = 128) leprosy patients (LP+/STH−) that had OD values ranging from 0.0245 to 0.6084. Among the individuals without leprosy (LP−), 15 participants had OD values ranging from 0.7120 to 0.9586 (LP−/STH+), significantly higher than the negative group (LP−/STH−), ranging from 0.0543 to 0.5080 (*p* = 0.0010). Among household contacts, 96 (54.5%) individuals were seropositive for STH, with OD values ranging from 0.6105 to 3.4183. Statistical differences between the groups are shown in Table 2.

**TABLE 2:** Comparison of optical density (OD) readings for IgG serology between groups.

Groups	*n*	OD range	Median	Comparative groups		*p*
LP−/STH−	43	(0.0543–0.5080)	0.1588	LP−/STH−	LP−/STH+	< 0.0001
LP−/STH+	15	(0.7120–0.9586)	0.8241		LP+/STH−	0.0139
LP+/STH−	128	(0.0245–0.6084)	0.4037		LP+/STH+	< 0.0001
LP+/STH+	123	(0.6103–4.2584)	0.8407		HHC/STH+	< 0.0001
HHC/STH−	80	(0.0735–0.6072)	0.3942	LP−/STH+	LP+/STH−	< 0.0001
HHC/STH+	96	(0.6105–3.4183)	0.8763		HHC/STH−	< 0.0001
				LP+/STH−	LP+/STH+	< 0.0001
					HHC/STH+	< 0.0001
				LP+/STH+	HHC/STH−	< 0.0001

Abbreviations: CV, coefficient of variation; HHC/STH− and HHC/STH+, household contacts group (negative and positive); LP/STH−, leprosy group with STH‐negative OD; LP/STH+, leprosy group with STH‐positive OD; SD, standard deviation; STH, soil‐transmitted helminths (negative [STH−] and positive [STH+]).

**FIGURE 1:**
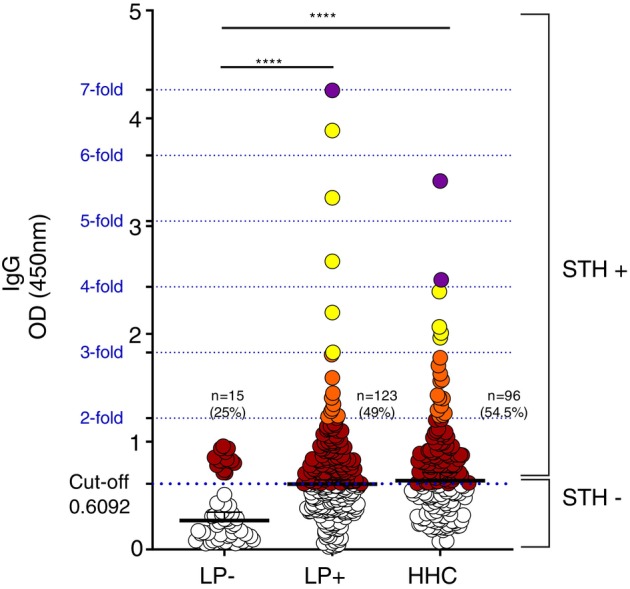
Serum levels of IgG antibodies against STH peptides in leprosy patients (LP+) and household contacts (HHC) compared with negative‐leprosy individuals (LP−). Optical density (OD) readings at 450 nm indicate IgG reactivity to a pool of immunogenic peptides from five STH species. Measures were performed in serum samples of (LP−) *n* = 58; (LP+) *n* = 251 and (HHC) *n* = 176. The IgG levels were quantified using ELISA as described in the Materials and Methods section. IgG levels in each study group were compared with those in LP− using the Kruskal‐Wallis test. The IgG seronegative (STH−) (white circle) and IgG seropositive (STH+) (coloured circles) individuals are demonstrated on the right side of the graph. The dashed orange line represents the cutoff value (0.6092). The percentage and number of individuals above the cutoff within each group are indicated in parentheses. Statistical differences between the groups were considered at *p* < 0.05. ****p < 0.0001.

Figure 1 illustrates the levels of specific serum IgG antibodies in response to a pool of immunogenic peptides derived from five STH species. OD at 450 nm represents IgG reactivity in leprosy‐negative individuals (*n* = 58), leprosy patients (*n* = 251) and household contacts (*n* = 176). The orange dotted line indicates the cutoff value for IgG positivity (OD = 0.6092). Among leprosy patients, 123 (49%) exhibited IgG positivity to STH antigens (LP+/STH+), whereas 128 (51%) were seronegative (LP+/STH−). Within the LP+/STH+ group, 9 individuals (7.3%) displayed IgG levels at least 2‐fold the cutoff value (orange), whereas 5 (4.1%) had values exceeding 3‐fold the reference threshold (yellow). One individual (0.8%) presented values 7‐fold above the cutoff (purple). In the leprosy‐negative group, 15 individuals (25.8%) tested IgG‐positive (LP−/STH+), serving as the positive control for the assay. Among the 176 household contacts, 96 (54.5%) (HHC/STH+) were seropositive for STH. Additionally, 13 (7.4%) presented IgG levels at least 2‐fold the cutoff, 4 (2.3%) had values 3‐fold above and 2 (1.1%) exhibited levels four‐ and five‐fold above the cutoff (green).

**FIGURE 2:**
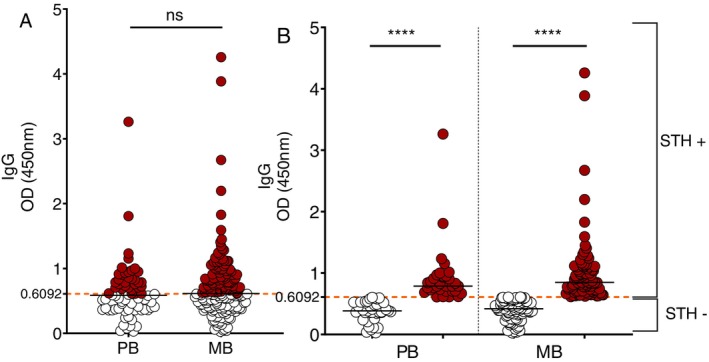
IgG serology to helminths in leprosy patients stratified by operational classification and helminth serostatus. (A) Scatter dot plots show optical density (OD) values at 450 nm for anti‐helminth IgG detected by ELISA in paucibacillary (PB; *n* = 72) and multibacillary (MB; *n* = 179) leprosy patients. No significant difference was observed between the groups (*p* = 0.5775) using Fisher's exact test. (B) When patients were stratified by both operational classification and helminth serostatus, significantly higher OD values were detected in PB STH+ compared to PB STH−, and in MB STH+ compared to MB STH− (****p < 0.0001) using the Kruskal‐Wallis test with Dunn's post hoc for multiple comparisons within groups. The orange dashed line indicates the positivity cut‐off (OD = 0.6092).

For subsequent analyses, leprosy patients were stratified into PB (*n* = 72) and MB (*n* = 179) groups. The comparison of helminth IgG seropositivity between these groups showed no statistically significant association using Fisher's exact test (*p* = 0.5775). Seropositivity was observed in 45.8% of PB and 50.3% of MB patients; however, the relative risk (RR = 1.089; 95% CI: 0.8286–1.393) and odds ratio (OR = 1.195; 95% CI: 0.6922–2.091) suggested a slightly higher risk and odds of seropositivity among MB patients. Mean OD values were also similar between groups, with 0.6286 ± 0.4381 in PB and 0.7055 ± 0.5174 in MB patients, reinforcing the lack of difference in overall IgG levels to helminths (Figure 2A). However, when patients were simultaneously stratified by both operational classification and helminth serostatus, Dunn's multiple comparisons test revealed highly significant differences within each group. Specifically, IgG reactivity was significantly higher in STH+ individuals compared to STH− within both PB (adjusted *p* < 0.0001) and MB (*p* < 0.0001) groups (Figure 2B).

**FIGURE 3:**
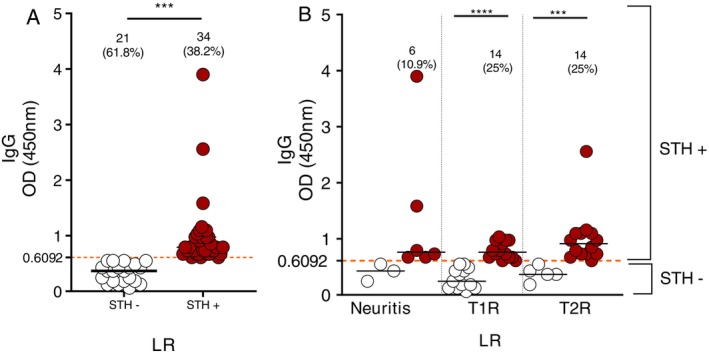
IgG serum levels detected in leprosy patients with inflammatory reactions, comparing the seronegative and seropositive for STH immunogenic peptides. (A) OD values comparing leprosy patients with negative (STH−) *n* = 21 and positive (STH+) *n* = 34 serology (*p* < 0.001). (B) Among LR patients with STH positivity, 6 (10.9%) presented with neuritis, 14 (25%) had a type 1 reaction (T1R) (*p* < 0.0001) and 14 (25%) had a type 2 reaction (T2R) (*p* < 0.0001). The dashed orange line indicates the cutoff value for seropositivity (≥ 0.6092 nm). The Kruskal‐Wallis test was used to compare STH seropositivity between groups. Significance was considered at *p* < 0.05. ***p < 0.001; ****p < 0.0001.

Among 251 leprosy patients, 55 (20.3%) had at least one type of inflammatory reaction episode. Of these, 34 (61.8%) had positive serology for the STH peptides (LR+/STH IgG+), whereas 21 (38.2%) had negative serology for STH antigens (LR+/STH IgG−) (Figure 3A). Among the LR+ patients, 9 (16.4%) had neuritis, with 6 (10.9%) of these being STH+; 27 (49.1%) had T1R, of which 14 (25%) were STH+; and 19 (34.5%) had T2R, with 14 (25%) testing positive for STH IgG (Figure 3B).

## 4 Discussion

Soil‐transmitted helminths are intestinal nematodes, mainly *Ascaris* sp., *Trichuris trichiura, Strongyloides stercoralis* and the hookworm species, that is, *Necator americanus* and *Ancylostoma duodenale*. These parasitic diseases are globally prevalent, primarily affecting impoverished populations or those with limited access to healthcare services. According to the 2019 Global Burden of Disease (GBD) Study, STHs account for millions of DALYs worldwide; although their burden has decreased in recent decades [27]. The persistent burden of STH infections, driven by social, economic and environmental factors, is often aggravated by their co‐occurrence with diseases like leprosy, as shown by the geospatial epidemiologic map of leprosy [5] and STHs [4].

A recent meta‐analysis reported STH prevalence of approximately 20% across thirteen Latin American countries [28]. In Brazil, national surveys reveal the presence of STH infections in all regions, with the highest prevalence observed in the Northeast, ranging from 2% to 36% between 2010 and 2015, and reaching up to 70% among school‐aged children [29]. Among states with municipalities up to 500,000 inhabitants, Sergipe and Minas Gerais, both included in our study, reported notable positivity rates of 6.62% and 5.81%, respectively [30]. Moreover, despite extensive deworming programs, a recent investigation found a 12% prevalence of intestinal parasites among schoolchildren in Sergipe [31].

The epidemiological overlap extends to immunological interactions between these infections. For instance, a study in southeastern Brazil demonstrated a positive association between co‐endemic leprosy and schistosomiasis, particularly with MB leprosy, possibly reflecting shared environmental risk factors and immunological phenomena such as chronic inflammation, immune modulation or antigenic cross‐reactivity between *M. leprae* and *S. mansoni* [21].

In our study, we observed high serological levels of anti‐IgG for STH among leprosy patients, with most cases being MB. We assume that using immunogenic peptides in the ELISA technique for IgG detection allowed us to identify an almost 50% seroprevalence based on OD readings in leprosy patients. Additionally, household contacts exhibited a similarly high IgG positivity rate of 56.5%, underscoring the role of shared environmental exposure within domiciliary settings. These findings align with well‐established risk factors for helminth infections, including inadequate sanitation, limited access to treated water, overcrowding, low socioeconomic status and proximity to domestic animals [32, 33, 34].

Despite the shared exposure, variability in antibody levels was observed regardless of leprosy operational classification, suggesting that environmental factors alone do not fully explain immune response differences. Helminths are known to induce a Th2‐biased immune response and to promote an immunoregulatory environment characterised by elevated production of IL‐10 and TGF‐β [35, 36], which can suppress the Th1‐mediated immunity essential for controlling *M. leprae* infection. This has been previously observed in lepromatous leprosy patients with intestinal helminths, who showed reduced intracellular levels of interferon‐γ [14, 35, 36]. Although no statistically significant direct association was found between STH seropositivity and leprosy classification, intragroup differences and risk estimates suggested a trend towards higher STH seropositivity among MB patients. Altogether, these observations indicate the possibility that host genetics and immune regulatory mechanisms may influence the complex interplay between helminth infections and leprosy.

When focusing on leprosy reactional episodes, our data revealed an IgG seropositivity rate for helminths of approximately 62%, despite some missing clinical data. Notably, a subset of patients showed markedly elevated antibody levels: two patients with neuritis had IgG levels 2.5‐ and 6.4‐fold above the cutoff and one patient with a T2R had a 4.2‐fold increase. Prior studies have reported diverse findings regarding the association between STH infections and leprosy reactions. For example, an Indonesian study found a significant association between STH infection and type 2 leprosy reactions, reporting a prevalence of 72.7% among patients with a history of reactions (*p* = 0.018) [17]. In contrast, a study from Nepal suggested an inverse relationship, possibly related to prior deworming interventions that may interrupt STH‐mediated immune dysregulation and consequently reduce the occurrence of leprosy reactions [13]. However, neither study included immunological analyses, which limit the strength of their conclusions.

Other comorbidities also contribute to leprosy reactions. A Brazilian study identified erythema nodosum leprosum (ENL), characterised by an immune complex‐mediated reaction, as the most frequent complication in MB patients, with coinfections such as oral, urinary, sinus or hepatitis infections increasing ENL risk by over twofold [37]. ENL is a recurrent and debilitating condition characterised by inflammation, fever, arthralgia and myalgia, often complicating treatment adherence and outcomes [38, 39]. A severe T2R case in Indonesia associated with *Trichuris* and hookworm infections highlighted the clinical importance of detecting and treating helminth co‐infections in leprosy; the patient's symptoms resolved only after anthelmintic therapy, emphasising the need for integrated management [15].

While co‐infections like HIV, viral hepatitis [40], bacterial infections [41] and parasitic co‐infections [19] have been recognised as risk factors for severe leprosy and reactional states, the role of helminths remains insufficiently explored. Understanding how helminth infections might influence the progression of leprosy and the oscillations in immune response could be crucial, as some drug therapies may trigger severe immune responses, demanding careful consideration in therapeutic strategies [38]. The immune mechanisms behind leprosy reactions are complex. T1R is generally associated with a strong cell‐mediated immune response, whereas T2R relates to immune complex deposition and a predominantly humoral response. This response may involve various cytokine patterns, including Th1, Th2, Th9, Th17, Th22 and Treg [8]. Helminth infections are known to suppress the Th1 immune response, which could theoretically increase the risk of T2R and reduce the risk of T1R [38, 42].

Traditional diagnostic methods for helminths, especially microscopy‐based techniques, often lack sensitivity in low‐prevalence settings [43, 44]. In this regard, bioinformatics approaches have advanced the field by predicting specific immunogenic epitopes from STH proteomes, as applied in our study. These tools enable the detection of specific IgG antibodies with greater sensitivity and specificity, minimising cross‐reactivity and improving diagnostic accuracy [26]. We believe that incorporating such epitope‐based serology could serve as an initial screening for STH exposure in leprosy patients, guiding subsequent confirmatory tests such as Kato‐Katz for parasite identification and guiding pharmacological treatment.

On the other hand, monitoring co‐infections supports more effective public health strategies for leprosy and helminth control. Detecting STH seropositivity in leprosy contacts demonstrates ongoing helminth exposure, suggesting co‐infection as a potential risk factor for immunological complications. An ecological study in 521 Brazilian municipalities linked access to filtered water and adequate school sanitation with lower rates of ascariasis, trichuriasis and hookworm infections [45], revealing that simple interventions are critical for STH control.

Despite its strengths, this study has limitations. Incomplete data on demographic variables, clinical classification, socioeconomic status, prior anthelmintic treatment and reactional episodes restricted more detailed adjusted analyses, potentially reducing discrimination between PB and MB groups and limiting the detection of significant differences in relative risk and odds ratios. To minimise confounding, we employed standardised protocols, ensured accurate diagnosis by qualified physicians, randomised samples during experimental procedures, stored sera at –80°C, and included well‐characterised control groups. Geographic representation was balanced across states to reduce bias; however, residual confounding due to unmeasured variables or the cross‐sectional design cannot be fully excluded. Despite these constraints, stratified analyses revealed significant differences in IgG reactivity linked to leprosy reactions, supporting the biological relevance of helminth exposure.

Additionally, the cross‐sectional design limits the ability to establish causal relationships between helminth co‐infections and leprosy outcomes. While IgG serology is effective for detecting exposure, it may not fully reflect active helminth infections, potentially leading to an underestimated prevalence. Future longitudinal studies incorporating comprehensive clinical data and direct parasitological assessments are needed to strengthen these findings and further elucidate the impact of helminth infections on leprosy progression and immune responses.

Overall, the immune imbalance from the interaction between leprosy and helminth co‐infections significantly affects the immune response, thereby impairing the body's ability to control *M. leprae* infection [40]. Chronic helminth infections also alter the intestinal microbiota, influencing systemic immune responses, potentially exacerbating inflammatory reactions, and complicating treatment [46, 47]. Addressing these co‐infections through improved diagnostics, targeted therapies and identification of immunological biomarkers is relevant for advancing leprosy management.

Our study adds valuable evidence to a topic that remains rarely explored, emphasising the importance of considering helminth exposure in the clinical follow‐up of leprosy patients. By applying an epitope‐based serological approach developed in our laboratory, we were able to generate insights into the complex immunological interplay that may influence disease progression and reactional episodes. We hope that these findings encourage further research and foster practical strategies to integrate parasite monitoring into leprosy care, contributing to more comprehensive patient management.

## 5 Conclusion

IgG seropositivity for soil‐transmitted helminths (STH) was detected in approximately half of the leprosy patients and their household contacts, indicating shared exposure within domiciliary environments. Stratified analyses showed significant differences in IgG levels between STH‐seropositive and seronegative individuals within both PB and MB groups, highlighting immune response variations beyond operational classification. Patients experiencing inflammatory reactions presented higher anti‐STH IgG levels compared to seronegative counterparts, suggesting a potential role of helminth exposure in reactional episodes. These findings emphasise the importance of screening for helminth co‐infections in leprosy management. Early detection and treatment of helminth infections may mitigate immune dysregulation and reduce inflammatory complications, supporting integrated approaches to improve patient outcomes. Further studies are needed to clarify the immunological mechanisms underlying these interactions.

## Conflicts of Interest

The authors declare no conflicts of interest.

## Supporting information


Figure S1:
