# Supplementary figures and images for: Serological Evidence of Soil‐Transmitted Helminth Infections as a Potential Risk for Severity in Leprosy Patients
Source: Trop Med Int Health. 2025 Aug 16;30(10):1115–23. doi: 10.1111/tmi.70020 (PMC12501561; doi:10.1111/tmi.70020)

**Figure S1**

**
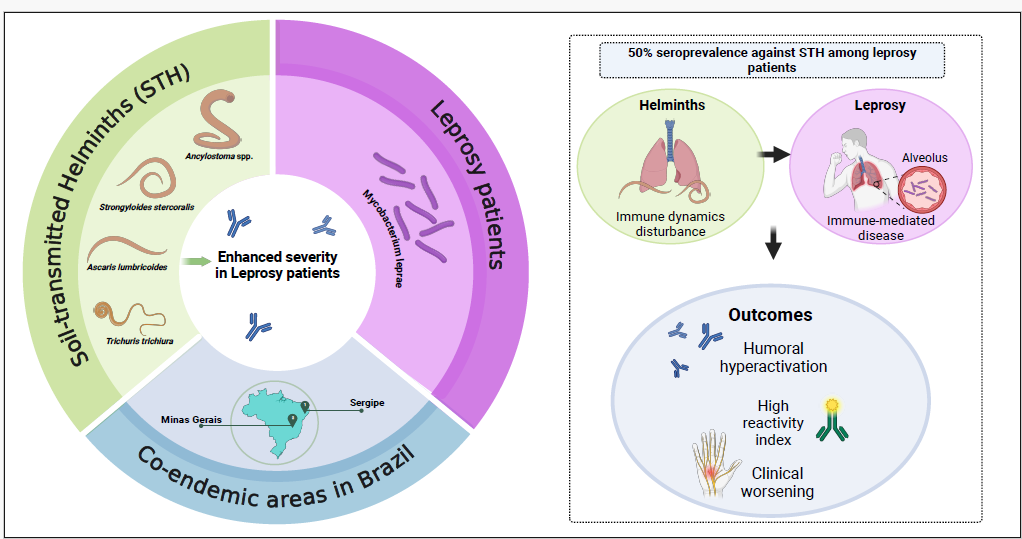
**

*Created in BioRender*

Supplement: Supplementary file 1 — Figure S1: [file TMI-30-1115-s001.docx]
